# A-CRNN-Based Method for Coherent DOA Estimation with Unknown Source Number

**DOI:** 10.3390/s20082296

**Published:** 2020-04-17

**Authors:** Yuanyuan Yao, Hong Lei, Wenjing He

**Affiliations:** 1 Department of Space Microwave Remote Sensing System, Aerospace Information Research Institute, Chinese Academy of Sciences, Beijing 100190, China; yaoyuanyuan17@mails.ucas.ac.cn (Y.Y.); hewenjing17@mails.ucas.ac.cn (W.H.); 2 School of Electronic, Electrical and Communication Engineering, University of Chinese Academy of Sciences, Beijing 100039, China

**Keywords:** direction-of-arrival (DOA) estimation, convolutional-recurrent neural network, Toeplitz matrix reconstruction, colored Gaussian noise, coherent sources

## Abstract

Estimating directions of arrival (DOA) without knowledge of the source number is regarded as a challenging task, particularly when coherence among sources exists. Researchers have trained deep learning (DL)-based models to attack the problem of DOA estimation. However, existing DL-based methods for coherent sources do not adapt to variable source numbers or require signal independence. Herein, we put forward a new framework combining parallel DOA estimators with Toeplitz matrix reconstruction to address the problem. Each estimator is constructed by connecting a multi-label classifier to a spatial filter, which is based on convolutional-recurrent neural networks. Spatial filters divide the angle domain into several sectors, so that the following classifiers can extract the arrival directions. Assisted with Toeplitz-based method for source-number determination, pseudo or missed angles classified by the estimators will be reduced. Then, the spatial spectrum can be more accurately recovered. In addition, the proposed method is data-driven, so it is naturally immune to signal coherence. Simulation results demonstrate the predominance of the proposed method and show that the trained model is robust to imperfect circumstances such as limited snapshots, colored Gaussian noise, and array imperfections.

## 1. Introduction

Direction-of-arrival (DOA) or spatial spectrum estimation is one of the most important content in array signal processing, which has been widely applied in navigation, acoustics, electronic reconnaissance [1,2], etc. The past few decades have witnessed the emergence of numerous high-resolution DOA estimation algorithms, which can break through the Rayleigh limit [3]. However, the performance remains to be further improved in some non-ideal situations, such as multi-path effect, limited snapshots, and colored noise.

Traditional solutions to high-resolution DOA estimation are well-known subspace-based algorithms, sparse representation-based methods, etc. Taking one of the most representative subspace-based techniques, for instance, multiple signals classification (MUSIC) [4], it conducts signal subspace decomposition to search for steering vectors approximately orthogonal to noise subspace, and the corresponding angles are considered as arrival directions. These MUSIC-based algorithms [5,6,7,8] can achieve considerably high angular-resolution, but usually require the given signal number, signal independence, and adequate snapshots. Fortunately, Ciuonzo [9] has proposed the approach of locating scatterers with unknown number on the foundation of cell-by-cell processing, which provides a new perspective to improve basic MUSICs. Another common improvement for coherent sources is using spatial smoothing [10] to form the full-ranked source covariance matrix, so that the MUSIC algorithm can be successfully applied. During the past two decades, direction finding by sparse reconstruction aims at minimizing the difference between data covariance matrix and the sparsely reconstructed one, which can be formulated into a convex optimization problem [11,12,13]. In this methodology, DOAs can be estimated on-grid or off-grid, and the source number is not compulsorily required. However, the procedure of finding the sparse solution and detecting the peaks located in the sparse spatial spectrum may pose a threat to pratical cases in terms of computation complexity with the increasing of the matrix sizes.

To overcome the difficulties mentioned above, learning-based methods have been introduced to find the directions. These robust and flexible techniques have been proven an excellent tool in synthetic and realistic data sets [14,15,16]. Dating back to the 1990s, the radial-basis function neural network (RBFNN) was adopted to learn nonlinear mappings from covariance matrices to spatial spectrums [15]. The key benefit arising from the RBFNNs is that they are in common use for correlated as well as uncorrelated signals. However, for small angular spacing, RBFNNs may be tough to separate these angles, while deep learning (DL) networks are capable to work well. In recent years, deep convolutional neural networks (CNN) [17] and recurrent neural networks (RNN) [18] have been applied in DOA estimation. Liu et al. took autoencoders and full-connected layers to build the deep neural network (DNN) framework, which could well resist several kinds of array imperfections. Following the one-vs-all classification guideline, DNN models in [19] were able to detect the number of sources. The algorithm has brought many improvements, however it is merely applicable to independent sources for using linear autoencoders. Lately, RNN has been approved of an excellent structure in time-series processing [20]. Perotin et al. stacked RNNs up to convolution layers to locate the sources with a given number, and the database formed by realistic acoustic signals had represented the effectiveness. However, once the source number changed, the model has to be trained again.

Present DNN-based DOA estimation models mostly ask for signal independence or a prior knowledge of source number. Moreover, a large percentage of studies concentrate on acoustic signals or other special application scenarios [16,21]. Motivated by this, we propose a new DL-based method to realize stable spatial spectrum recovery appropriate for varying signal numbers in this paper. The hierarchical DNN-based method [19] and conventional spatial-smooth MUSIC (SS-MUSIC) algorithm [10] are chosen for comparison. Simulations on uniform linear array (ULA) show that the proposed method shows great advantage in terms of mean absolute error (MAE) and has good adaptability to untrained situations. The main contributions made in our work are concluded as below.

- We design a framework based on alternate convolutional-recurrent neural network (A-CRNN), which is feasible to DOA estimation regardless of the signal coherence.
- The scheme that source number is jointly determined by multi-label estimators and reconstructed Toeplitz matrices is employed, which greatly improves the performance of direction finding.
- Considering the class and label imbalances happening during the training of sub-networks, we adopt Focal loss [22] and data augmentation to reduce the negative effects.
- Colored noise and other array imperfections are considered, which validates the robustness for potential practical systems.

The rest of the paper is organized into five parts. The related mathematical foundations are given in Section 2. Section 3 describes the details of modeling A-CRNNs for DOA estimation. In Section 4, some simulations and comparisons are conducted. Finally, Section 5 draws a conclusion of the whole work.

Before the description, the mathematical notations we used are explained. Boldface letters in upper case and lower case, respectively, denote matrices and column vectors. Scalars are signed with lowercase letters. R and C mean the sets of real numbers and complex numbers. The operations of inverse, transpose, conjugate transpose and complex conjugate are expressed by ${\left(\;\cdot \;\right)}^{-1}$, ${\left(\;\cdot \;\right)}^{T}$, ${\left(\;\cdot \;\right)}^{*}$ and ${\left(\;\cdot \;\right)}^{H}$. E{a} represents the expectation of *a* and ∥·∥ denotes the Euclidean norm.

## 2. Problem Formulations

In this section, we first discuss the mathematical foundations of array receiving model for coherent signals. After that, a Toeplitz matrices-based method for source counting is introduced. To this end, we describe the multi-label classification (MLC) [23] strategy employed in the proposed DL, which fully meets the requirement of multiple directions finding.

### 2.1. Array Signal-Receiving Model

**Figure 1:**
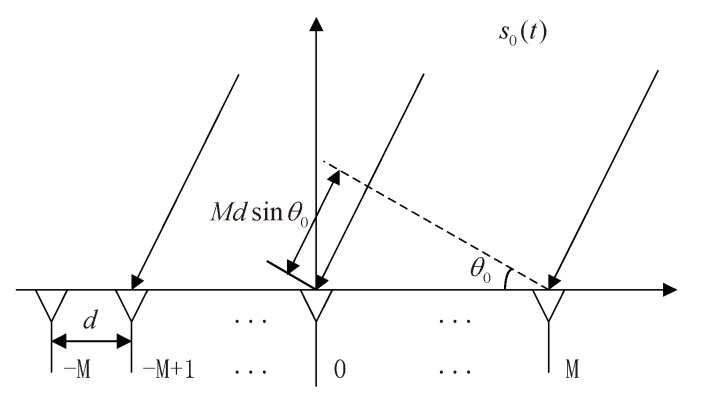
Uniform plane waves received by a (2M+1)—element ULA.

Consider *P* far-field narrowband signals with a common center frequency $f_{0}$ impinging to a ULA as illustrated in Figure 1, the sensor number of which is (2M+1). We assume that sensors are isotropic with equal inter-element space denoted by *d*, (M+1)≥P, and K(K≤P) signals are coherent. Let the first signal $s_{0}\left(t\right)$ as the reference without loss of generality, and the rest coherent sources can be expressed as

(1) $$s_{k}\left(t\right)=\alpha_{k}e^{j\beta_{k}}s_{0}\left(t\right),\;k=1,\cdots ,K-1$$

where the complex constant $\rho_{k}=\alpha_{k}e^{j\beta_{k}}$ is the correlation coefficient between $s_{0}\left(t\right)$ and $s_{k}\left(t\right)$. Then, array output of the *m*th element becomes

(2) $$\begin{array}{cc} x_{m}\left(t\right) & =\sum_{i=0}^{P-1}e^{-j\frac{2\pi f_{0}}{c}\left(md\sin\theta_{i}\right)}s_{i}\left(t\right)+n_{m}\left(t\right)\\ \; & =s_{0}\left(t\right)\sum_{i=0}^{K-1}\rho_{i}e^{-j\frac{2\pi f_{0}}{c}\left(md\sin\theta_{i}\right)}+\sum_{i=K}^{P-1}e^{-j\frac{2\pi f_{0}}{c}\left(md\sin\theta_{i}\right)}s_{i}\left(t\right)+n_{m}\left(t\right)\\ \; & m=-M,\cdots ,0,\cdots ,M \end{array}$$

where $\theta_{i}$ is the *i*th arrival direction, *c* stands for the velocity of light in vacuum, and $n_{m}\left(t\right)$ represents the additive white Gaussian noise (AWGN) with zero mean-value. Let d=λ/2, where λ is the wavelength of carriers, and we can write the observation signals in a vector manner as

(3) $$x\left(t\right)={\left[x_{-M}\left(t\right),\ldots ,x_{0}\left(t\right),\cdots ,x_{M}\left(t\right)\right]}^{T}=As\left(t\right)+n\left(t\right)$$

where $s\left(t\right)={\left[s_{0}\left(t\right),\ldots ,s_{P-1}\left(t\right)\right]}^{T}$ is the source signal vector, $n\left(t\right)={\left[n_{-M}\left(t\right),\ldots ,n_{0}\left(t\right),\ldots ,n_{M}\left(t\right)\right]}^{T}$ represents noise vector, and A is steering matrix with

(4) $$A=\left[\begin{array}{cccc} e^{jM\pi \sin\theta_{0}} & e^{jM\pi \sin\theta_{1}} & \cdots & e^{jM\pi \sin\theta_{P-1}}\\ e^{j\left(M-1\right)\pi \sin\theta_{0}} & e^{j\left(M-1\right)\pi \sin\theta_{1}} & \cdots & e^{j\left(M-1\right)\pi \sin\theta_{P-1}}\\ \vdots & \vdots & \; & \vdots \\ 1 & 1 & \ddots & 1\\ \vdots & \vdots & \; & \vdots \\ e^{-jM\pi \sin\theta_{0}} & e^{-jM\pi \sin\theta_{1}} & \cdots & e^{-jM\pi \sin\theta_{P-1}} \end{array}\right]$$

### 2.2. Source Number Determination

In pratical spatial spectrum recovery, we will not be informed of the source number beforehand. Therefore, we embed an extra algorithm on the basis of Toeplitz matrices decomposition in our framework to detect the source number, which performs well whether signals are coherent or not.

Taking the *m*th row of the output covariance matrix $R=E\{x\left(t\right)x^{H}\left(t\right)\}$, we can reshape a Toeplitz matrix $R_{m}$ as below,

(5) $$R_{m}=\left[\begin{array}{cccc} r(m,0) & r(m,1) & \cdots & r(m,M)\\ r(m,-1) & r(m,0) & \cdots & r(m,M-1)\\ \vdots & \vdots & \ddots & \vdots \\ r(m,-M) & r(m,-M+1) & \cdots & r(m,0) \end{array}\right]=DS_{m}D^{H}+\sigma_{n}^{2}I_{M+1,m}$$

where $I_{M+1,m}\in R^{\left(M+1)\times (M+1\right)}$ with one on the *m*th diagonal and zero elsewhere. By conducting the eigen-decomposition on $R_{m}$, we can get $D=[d\left(\theta_{0}\right),\cdots ,d\left(\theta_{P-1}\right)]$ with

(6) $$d\left(\theta_{i}\right)={\left[1,e^{-j\pi \sin\theta_{i}},\cdots ,e^{-jM\pi \sin\theta_{i}}\right]}^{T}$$

and $S_{m}=$ diag$\left\{s_{m,0},\cdots ,s_{m,P-1}\right\}$ with

(7) $$s_{m,i}=\left\{\begin{array}{cc} P_{1,1}\rho_{i}^{*}\sum_{k=0}^{K-1}\rho_{k}e^{-jm\pi \sin\theta_{k}}, & \;i=0,\cdots ,K-1\\ P_{i,i}e^{-jm\pi \sin\theta_{i}}, & \;i=K,\cdots ,P-1 \end{array}\right.$$

(8) $$P_{k,i}=E\left\{s_{k}\left(t\right)s_{i}^{*}\left(t\right)\right\},\;k,l=0,K,\cdots ,P-1$$

We can find that $P_{k,i}\neq 0$ for k=i in Equation (8), regardless of the signal coherence. Thus, ∀i∈{0,⋯,P−1}, we have $s_{m,i}\neq 0$, which means that the rank of $S_{m}$ is *P* and totally unrelated to the coherence among sources. Therefore, we can conduct the eigenvalue decomposition on $R_{m}$ and then pick out *P* larger eigenvalues, which completes the source number estimation. Here, we omit to give the derivations from Equations (5)–(8), which can be seen in [24].

### 2.3. Multi-Label Classification

In this paper, we model the multi-source DOA estimation as a MLC problem [23]. Let $U=\{\theta_{0},\theta_{1}\;\cdots ,\theta_{N-1}\}$ denote a finite label set of discrete angles and X represent the input space. For a given input instance x∈X, we suppose that it holds the label set u, and elements of u mean the angle values to be estimated. We can define a corresponding binary vector $y={\left[y_{0},y_{1},\cdots ,y_{N-1}\right]}^{T}$ to represent the grid directions with

(9) $$y_{n}=\left\{\begin{array}{cc} 1, & \theta_{n}-\frac{\Delta \theta }{2}\leq \theta <\theta_{n}+\frac{\Delta \theta }{2}\\ 0, & otherwise \end{array}\right.\;n=0,1,\cdots ,N-1$$

where θ∈u and Δθ>0 is set to be the angular grid resolution.

From Equation (9), we can acquire the output space denoted by $Y={\left\{0,1\right\}}^{N}$. Supposing that (x,y) is an observation independently and identically generated from X×Y, the modeling of MLC can be described as a optimization problem below,

(10) $$\min_{f\in F}\frac{1}{N}\sum_{n=0}^{N-1}L\left(y_{n},f\left(x_{n}\right)\right)+\lambda J\left(f\right)$$

where F describes the hypothesis space of the classification model with F={f|y=f(x)}, L(·) stands for the multi-label loss function like *binary-crossentropy*, and λJ(f) is called the regularization or penalty term depicting the model complexity.

## 3. A-CRNN-Based DOA Estimation

**Figure 2:**
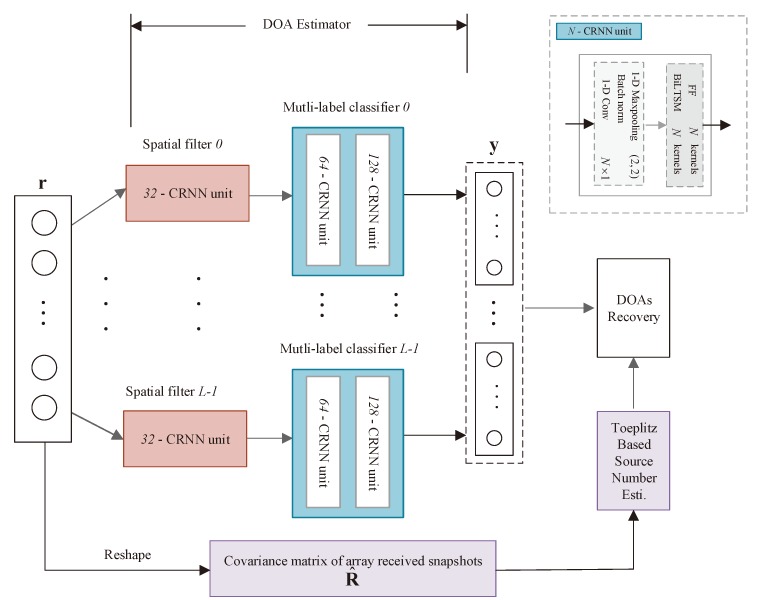
Framework of Toeplitz alternate convolutional-recurrent neural network (A-CRNN)-based coherent direction-of-arrival (DOA) estimation without knowing the source number.

To obtain higher angular resolution but not deteriorate the estimation accuracy, we design the A-CRNN architecture as illustrated in Figure 2, which is constituted of parallel pairs of spatial filter and multi-label classifier. Spatial filters allocate multiple directions to narrower angular sectors. Afterwards, the classifiers connected to them can give out the probabilities of discretized angles belonging to each sector. Simply following the one-vs-all guideline [19] to determine the source number is not reliable, which acts as setting a threshold of the output probability to identify the appearance of signals. Therefore, we embed the Toeplitz-based method to the networks for better detecting of source number, in order to avoid causing a few false and missed alarms of signal existence. Ultimately, source numbers can be estimated almost completely correct, and we can obtain the final DOA results by concatenating the results from all sectors.

For the goal of relieving the computational burdens, the proposed network receives input vector r following the guidelines of [19],

(11) $$\tilde{r}={\left[r_{0,1},r_{0,2},\cdots ,r_{0,2M+1},r_{1,2},r_{1,3},\cdots ,r_{1,2M+1},\cdots ,r_{2M,2M+1}\right]}^{T}\in C^{M(2M+1)\times 1}$$

(12) $$r=\frac{{\left[Real\left\{{\tilde{r}}^{T}\right\},Imag\left\{{\tilde{r}}^{T}\right\}\right]}^{T}}{∥\tilde{r}{∥}_{2}}\in R^{2M(2M+1)\times 1}$$

where $r_{m_{1},m_{2}}$ represents the $\left(m_{1},m_{2}\right)$th element of the estimated covariance matrix,

(13) $$\hat{R}=\frac{1}{S}\sum_{n=0}^{S-1}x\left(t_{s}\right)x^{T}\left(t_{s}\right)$$

where *S* is the number of snapshots.

### 3.1. Network Architecture

The proposed DOA estimation network is built by stacking different scales of the CRNN units as depicted in the upper right of Figure 2, which spontaneously establishes the alternative CNN and RNN structure. Unlike 2-dimensional (2-D) images, time series generated by vectorizing the covariance matrices form the 1-dimensional (1-D) input vectors. Furthermore, from Equation (2), it is obvious that the received data keeps invariant against the order of arrival angles. Thus, 1-D CNNs are appropriate for locally low-order feature extraction. However, the input data of the network is time-involved, and merely using of convolution operations can not give better fitting from the covariance data to angle values. Therefore, we are motivated to design the CRNN unit to construct the DOA estimation network.

Among various types of RNN layers, bidirectional long short-term memory (BiLSTM) and bidirectional gated recurrent units (BiGRU) [25] are two of the most popular structures. They can get access to inputs both in the forward and backward directions, which is benefit of exploring the time-dependence. In addition, they are capable to mitigate gradient vanishing. Even though the training of BiGRUs takes less time, they perform rather poorly regarding the tests to new data, which can be numerically verified in the later simulation section. In the long term, we decide to choose BiLSTMs as the recurrent neurons. Consequently, the *N*-CRNN unit is comprised of a convolution layer, a BiLSTM layer, and a fully-connected feed-forward (FF) [16] layer, all with *N* kernels.

#### 3.1.1. Spatial Filters

Each spatial filter shown in Figure 2 is formed with a 32-CRNN unit, deciding which classifier the inputs should be sent to. Compared to the linear autoencoder in [19], our filters allow coherent inputs and hold higher division accuracy. Choose L+1 angles $\theta_{0}<\theta_{1}<\cdots <\theta_{L}$, uniformly dividing the direction space into *L* intervals, which means $\theta_{1}-\theta_{0}=\cdots =\theta_{l}-\theta_{l-1}=\cdots =\theta_{L}-\theta_{L-1}$. Considering the signals arriving in the ULA from several directions of $\theta_{0},\theta_{1},\cdots ,\theta_{P-1}$, we can denote the output of the *l*th spatial filter as

(14) $$z_{l}=\left\{\begin{array}{cc} 1, & \theta_{l-1}\leq \theta_{p}<\theta_{l}\\ 0, & \mathrm{otherwise} \end{array}\right.\;\forall p\in \left\{0,1,\cdots ,P-1\right\};\;l=0,1,\cdots ,L-1.$$

#### 3.1.2. Multi-Label Classifiers

The second part of the fine-grained DOA estimator consists of *L* parallel A-CRNN-based classifiers. Larger *L* implies we need to train more sub-networks, whereas smaller *L* leads to degradation of estimation precision. Thus, we should consider a compromise when choosing the number of classifiers. All the multi-label classifiers illustrated in Figure 2 are constructed by stacking a 128-CRNN unit up to a 64-CRNN unit, which is then flattened to a dense layer as the output layer.

In order to make a MLC assignment, we sample the direction interval into *N* discrete angles shown below,

(15) $$\left\{\begin{array}{c} u_{l}=\left\{\theta_{l-1},\theta_{l-1}+\Delta \theta ,\cdots ,\theta_{l-1}+\left(N-1\right)\;\cdot \;\Delta \theta \right\}\\ N=\frac{\theta_{l}-\theta_{l-1}}{\Delta \theta } \end{array}\right.\;l=0,1,\cdots ,L-1$$

where Δθ is the angular resolution and $u_{l}$ denotes label set of the *l*th classifier. Let $y_{l}=y_{l,0},\cdots ,y_{l,n},\cdots ,y_{l,N-1}$ remarks output vector of the *l*th classifier. $y_{l,n}$, as described in Equation (9), expresses the probability if there exists an arrival direction which is equivalent to the *n*th element in $u_{l}$. It should be supplemented that the *l*th multi-label classifier will be triggered if and only if $z_{l}$ is equal to 1; otherwise, the output vector of it will be directly set to 0. Based on this strategy, the *l*th classifier does not have to take the DOAs outside of the interval $\left[\theta_{l-1},\theta_{l}\right)$ into consideration, which largely accelerates the training procedure.

### 3.2. Global DOA Estimation

Combining the outputs of *L* classifiers, the expected result of the whole network is written as

(16) $$\begin{array}{cc} y & ={\left[y_{0,0},\cdots ,y_{0,N-1},\cdots ,y_{ln},\cdots ,y_{L-1,0},\cdots ,y_{L-1,N-1}\right]}^{T}\\ \; & ={\left[y_{0}^{T},y_{1}^{T},\cdots ,y_{L-1}^{T}\right]}^{T}\in R^{LN\times 1} \end{array}$$

In particular, if the coming signals are all from the same sector, for instance, the *l*th sector, then the final outputs should be $y={\left[0_{N\times 1}^{T},\cdots ,0_{N\times 1}^{T},y_{l}^{T},0_{N\times 1}^{T},\cdots ,0_{N\times 1}^{T}\right]}^{T}$.

Supposing that the source number determined by means of the Toeplitz matrix-based method as aforementioned in Section 2.2 is $\hat{P_{0}}$. Then, $\hat{P_{0}}$ larger elements over the total L×N outputs from y in (15) are selected out and their corresponding subscripts constitutes a set denoted by

(17) $$D=\{d_{0},\cdots ,d_{p},\cdots ,d_{\hat{P_{0}}-1}\}$$

To further reduce the rate of determination failure, we set two threshold values $p_{min}$ and $p_{max}$, with $0<p_{min}<p_{max}<1$. On one hand, if $y_{d_{p}}<p_{min}$, $d_{p}$ will be removed from *D*. On the other, the index of the unselected $y_{ln}$ (15) with $y_{ln}>p_{max}$ will be added into the set *D*. At last, the source number will be updated to $\hat{P}$. We can gradually recover the directions $\left\{A_{0},\cdots ,A_{p},\cdots ,A_{\hat{P}-1}\right\}$ impinging to the ULA of multi-sources by

(18) $$A_{p}=\theta_{0}+\left(d_{p}\;\cdot \;\Delta \theta \right)+\left(y_{d_{p}}\;\cdot \;\Delta \theta \right),\;p=0,\cdots ,\hat{P}-1$$

where $\left(y_{d_{p}}\;\cdot \;\Delta \theta \right)$ accounts for the interpolation within the angular resolution Δθ.

**Figure 3:**
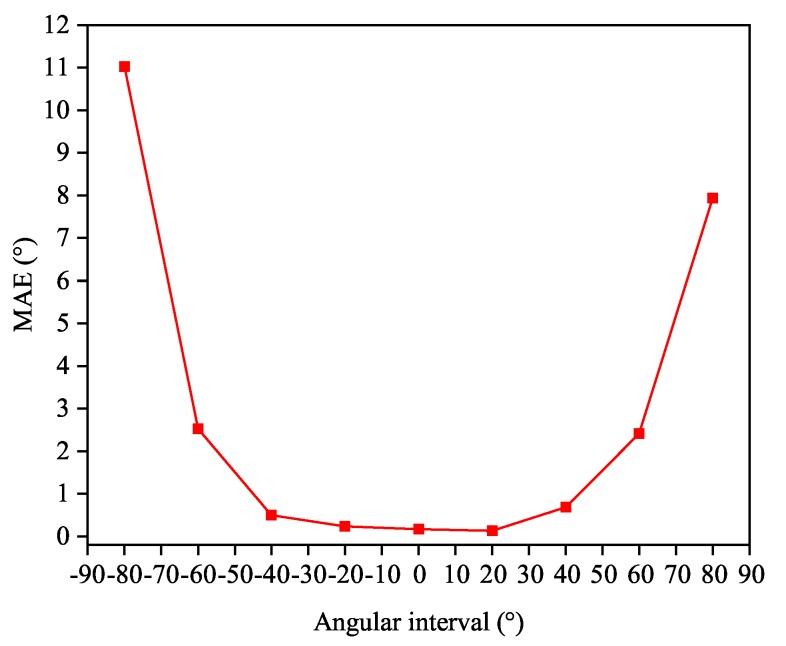
Mean absolute error (MAE) response curve of the multi-label classifiers in different sectors at the signal-to-noise (SNR) of 20 dB.

**Figure 4:**
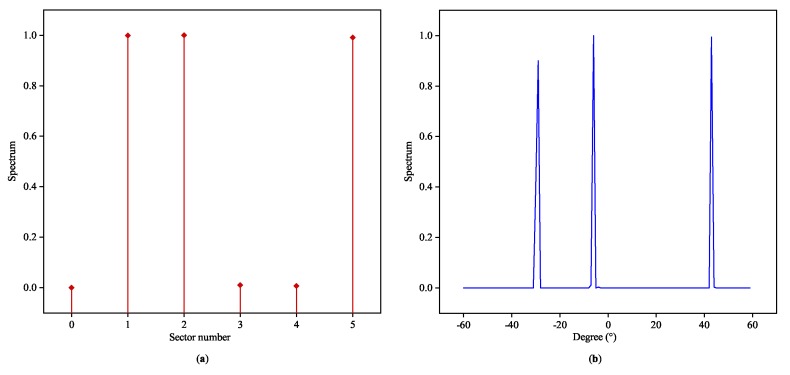
Recovered spatial spectrums for coherent signals from three different angular sectors: (**a**) outputs of the space filter to the signals from the 2th, 3th, and 6th sectors with the directions of (−28°,−5°,44°). (**b**) Outputs of the multi-label classifiers to the signals from the 1th, 2th, and 5th sectors with the directions of (−28°,−5°,44°).

**Figure 5:**
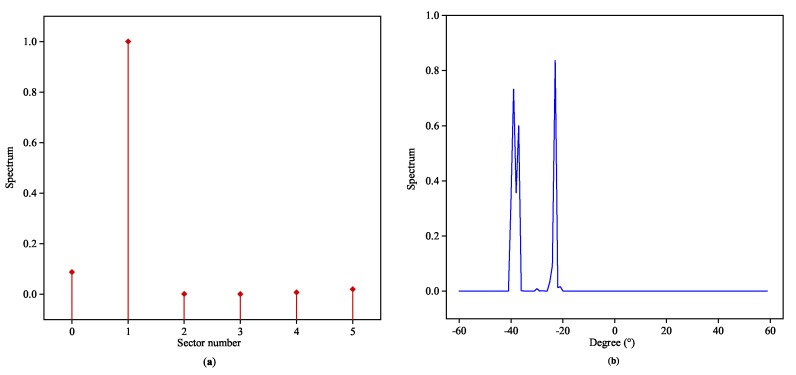
Recovered spatial spectrums for coherent signals from the same angular sector: (**a**) outputs of the space filter to the signals from the 2th sector with the directions of (−38°,−36°,−22°). (**b**) Outputs of the multi-label classifiers to the signals from the 2th sector with the directions of (−38°,−36°,−22°).

In the literature of classical estimation theory, the variance of phase estimation is proportional to $1/\cos^{2}\theta$, which indicates that the performance will sharply deteriorate when |θ| is close to 90° [26]. The proposed DOA classifier will also experience a relatively slight deline in accuracy with the angular sector approaching ±90°, and the corresponding MAE performance is depicted in Figure 3. We choose L=6 to uniformly split [−60°,60°). Figure 4 and Figure 5 exhibit two typical testing responses of the proposed Toeplitz A-CRNN model to three sources with the first two of them are coherent. Figure 4a depicts the dividing results of the sources from three different sectors with the directions (−28°,−5°,44°), and the concatenated output of six spatial filters $\left(z_{0},z_{1},\cdots ,z_{5}\right)$ is (0,1,1,0,0,1). Figure 5a gives the sector division of directions (−38°,−36°,−22°), which are all from the second sector. The reconstructed spatial spectrums are individually plotted in Figure 4b and Figure 5b.

## 4. Simulations and Discussions

In this section, numerical experiments are given and the results are discussed. First of all, the simulation conditions are stated. Then, we compare the coherent DOA estimation performances among the proposed method, hierarchical DNN-based algorithm [19], and traditional SS-MUSIC [10]. Finally, we test our Toeplitz A-CRNN algorithm in diverse untrained circumstances, which reveals well capability of generalization.

### 4.1. Simulation Settings

In the simulations, received data is hexadecimal quadrature amplitude modulation (QAM) signals generated from three narrow-band sources, with the first two of them coherent. Real and imaginary components of the coherent coefficients are randomly generated. Spacing between the array sensors is half of the wavelength, and other settings with respect to data preparation are listed in Table 1. We can construct the database by stochastically choosing 3 angular values from $(\theta_{max}-\theta_{min})\backslash \Delta \theta =120$ candidates, which totally produces $C_{120}^{3}=280,840$ samples. Different from the identical interval among directions in [19], our arbitrary sampling scheme more approaches the actual situation. To release the training loads, only one-sixth of the samples are reserved at random. We take 80%, 10%, and 10% of the instances for model training, validation, and testing.

**Figure 6:**
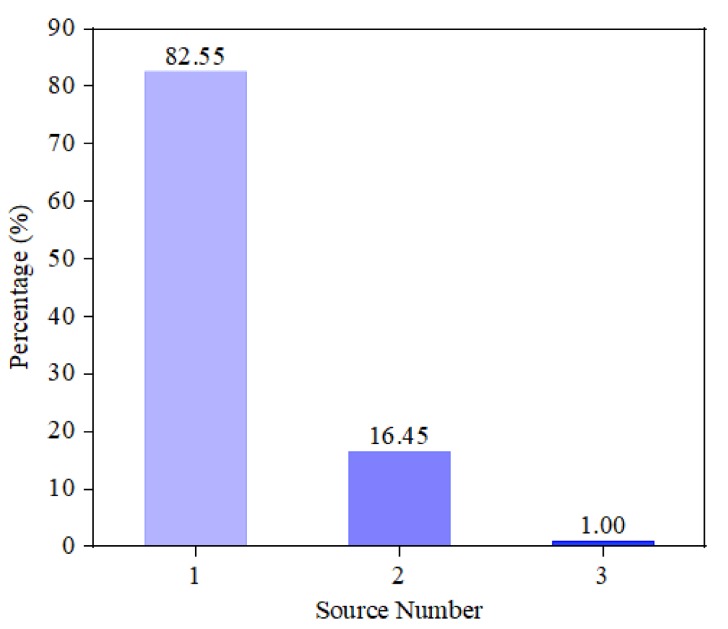
Source number distribution of the same sector.

Training settings concerning spatial filters and multi-label classifiers are enumerated in Table 2. In spatial filters, output layers are activated by the softmax function and other upper layers use relu function. For the training dataset to a spatial filter, only one-sixth of the samples belong to the positive class while others are negative for L=6, which can cause the class imbalance [27]. To settle this problem, we adopt Focal loss [22], which can guide the learning procedure inclining to the less positive samples. Similar to spatial filters, the popular relu is selected as the activation function to the layers of multi-label classifiers except for the last one, which is changed by sigmoid function to match the MLC loss described in (10). Instances in the same sector can be clustered to three categories distinguished by the source number from the current sector, which is possible to be 1, 2, or 3. The statistical distribution of the three cases is drawn in Figure 6. Observations containing two or three sources related to the same sector take proportions of 16.45% and 1.00%, respectively, yet the one-source case accounts for 82.55%, which forms the severe multi-label imbalance [28]. Hence, we re-sample the minority cases under two extra SNRs quite closing to 20 dB to balance the training dataset.

### 4.2. Comparison and Evaluation

**Figure 7:**
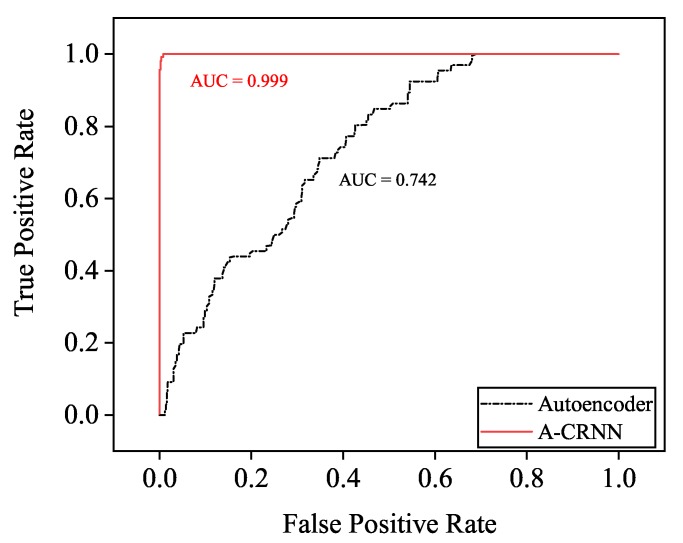
Receiver operating characteristic (ROC) curves of the autoencoder and the proposed A-CRNN spacial filter of independent two-source DOA estimation.

Recalling that autoencoders in [19] are only valid to independent signals, we extend our trained A-CRNN spatial filters to the scene of two independent sources to form a contrast, which reflects the generalization capability of our models to the independent scene as well. The larger area under receiver operating characteristic (ROC) curve [29] indicates the better performance of the binary classifier. Figure 7 plots the ROC curves of the spatial filters applied in the baseline and the proposed framework. The larger area-under-curve (AUC) means that the model will execute a correct classification in higher probability, and our spatial filter represents the superiority.

#### 4.2.1. Three-Source Testing under AWGN

In this part, we check the accuracies of coherent DOA estimation in AWGN environment. First, the SNR remains unchanged at 20 dB. Table 3 reports the results of five algorithms with absolute-error tolerances less than 1°, 4°, 7°, and 10° of the entire testing set. Moreover, average testing durations to different methods of a single instance are given. The best results are highlight in bold. The framework in [19] is constructed by multi-layer neural networks which are simply full-connected, and we briefly name it as FC-NN. FC-NN presents poor performance while having the best computative efficiency. It reflects that FC-NNs have limited feature extraction ability for multi-source DOA estimation. The proposed framework reaches the highest estimation accuracy and the testing time is less than the traditional SS-MUSIC algorithm, which indicates that our off-line trained models can relieve the computing burden without damaging the performance. Assisted by Toeplitz-based source-counting, 95.77% of the errors are suppressed smaller than <1°.

**Figure 8:**
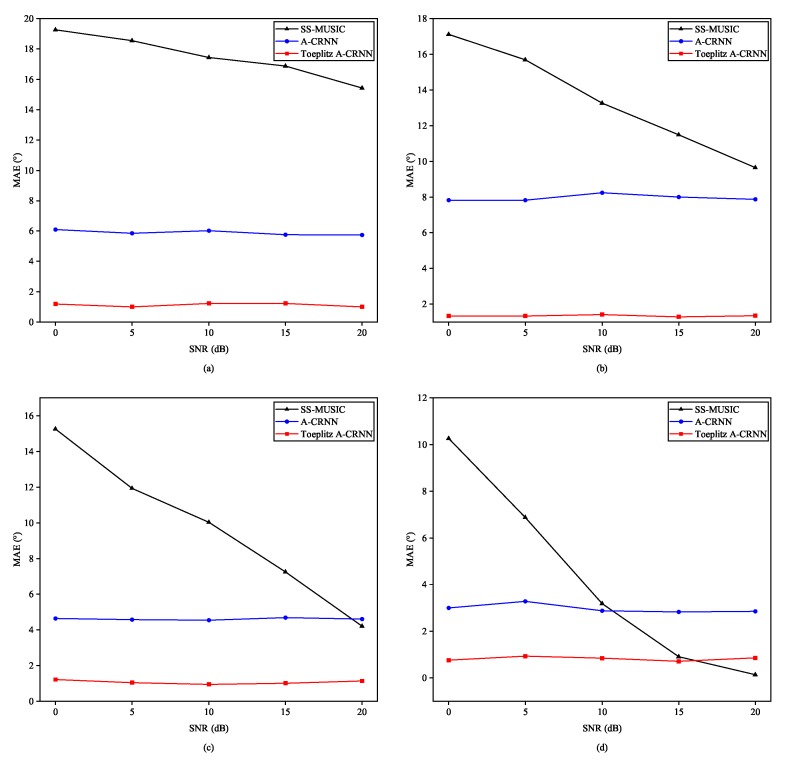
MAEs for direction combinations in the testing set with variable SNR levels and different angle intervals: (**a**) Angle interval = 3°. (**b**) Angle interval = 5°. (**c**) Angle interval = 8°. (**d**) Angle interval = 12°.

Second, testing results in variable levels of SNR are illustrated in Figure 8. We split the testing samples to four subsets with the minimum angle interval through three sources being 3°, 5°, 8°, and 12°. Seen from the subfigures in Figure 8, our Toeplitz A-CRNN method works better over the SNR levels when the angular distance is less than 8°. SS-MUSIC algorithm will outperform if both the SNR is high and the angle interval is significantly large, which are tough conditions in real applications.

**Figure 9:**
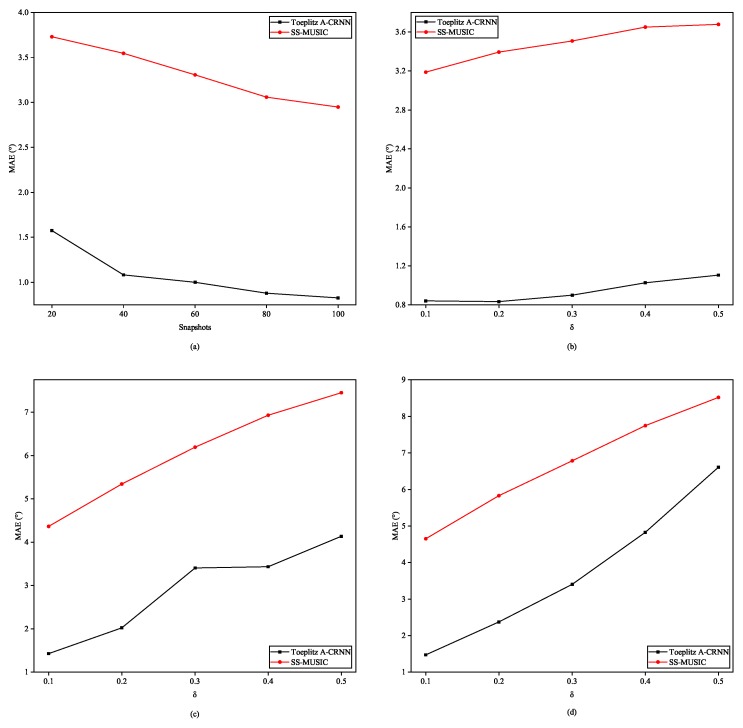
Performances of DOA estimation under imperfect circumstances with the SNR of testing data fixed at 20 dB: (**a**) different numbers of snapshots. (**b**) Sensor-gain inconsistence. (**c**) Combined gain inconsistence with biased sensor position. (**d**) Coexisting of gain inconsistence, position bias, and inter-sensor mutual coupling.

Finally, it commonly happens that we are not able to get a great enough number of snapshots. Moreover, several kinds of array imperfections always emerge, such as gain inconsistence, sensor-position bias, and inter-sensor mutual coupling, which have been well modeled in [19]. According to the authors of [19], the imperfect steering matrix can be rewritten as

(19) $$A=\left(I_{2M+1}+\delta \gamma_{\mathrm{mutual}}E_{\mathrm{mutual}}\right)\times \left(I_{2M+1}+\delta Diag\left(\gamma_{\mathrm{gain}}E_{\mathrm{gain}}\right)\right)\times A\left(\delta \gamma_{\mathrm{position}}e_{\mathrm{position}}\right)$$

where δ describes the intensity of deviations. Expressions of $E_{\mathrm{mutual}}$, $\gamma_{\mathrm{gain}}$, and $e_{\mathrm{position}}$ can be found in [19], which are omitted here. At this time, conventional direction finding algorithms often can not guarantee the effectiveness. Thus, we need to verify whether the proposed network trained on the ideal ULA can adapt to these pratical interferences. Spotted line charts in Figure 9 indicate that the proposed method remains powerful in learning the angle features in the contrast with traditional SS-MUSIC.

#### 4.2.2. Testing in Untrained Numbers of Source

**Figure 10:**
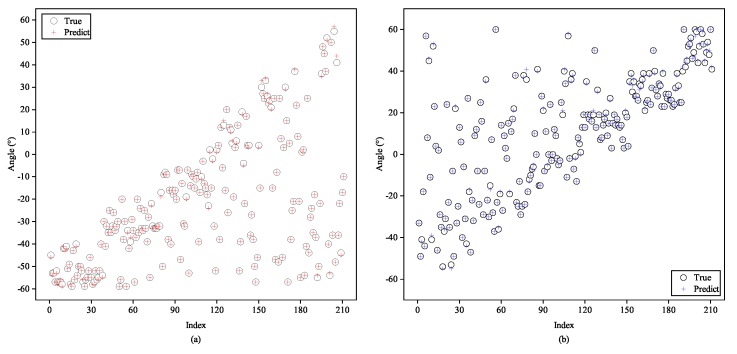
Testing of the proposed A-CRNN model to two-signal testing samples while trained in three-signal case: (**a**) the first signal and (**b**) the second signal.

**Figure 11:**
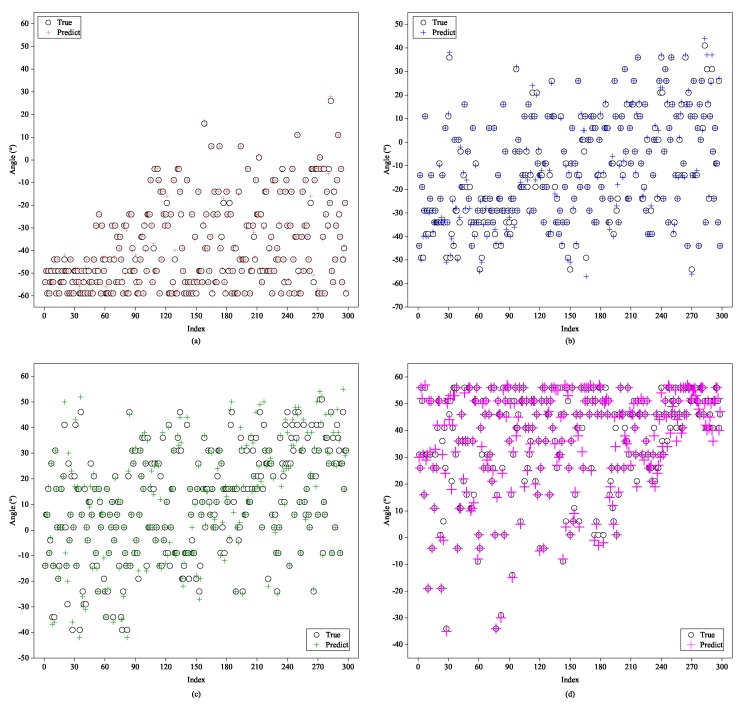
Testing of the proposed A-CRNN model to four-signal testing samples while trained in three-signal case: (**a**) the first signal, (**b**) the second signal, (**c**) the third signal, and (**d**) the fourth signal.

In order to examine the generalization ability of our framework to changeable number of sources, we form the two-signal and four-signal testing sets, which never appear in the training set, and the level of SNR is fixed at 20 dB. As individually illustrated in Figure 10 and Figure 11, subfigures of them depict DOA estimation performance to each one of the two or four signals. From the figures, we can see that most of the colored crosses denoting the predicted angles fall near the hollow black circles which stand for the ground-truth. Therefore, it is reasonable to deduce that the proposed method is self-adaptive to various numbers of arrival directions.

#### 4.2.3. Generalized to Colored Gaussian Noise

In practice, the noise is almost colored. Therefore, it is necessary to test the robustness of our trained models to colored Gaussian noise, which is simply simulated as [30]

(20) $$n_{c}\left(t\right)=n\left(t\right)+0.5n\left(t-1\right)$$

where n(t) denotes AWGN.

**Figure 12:**
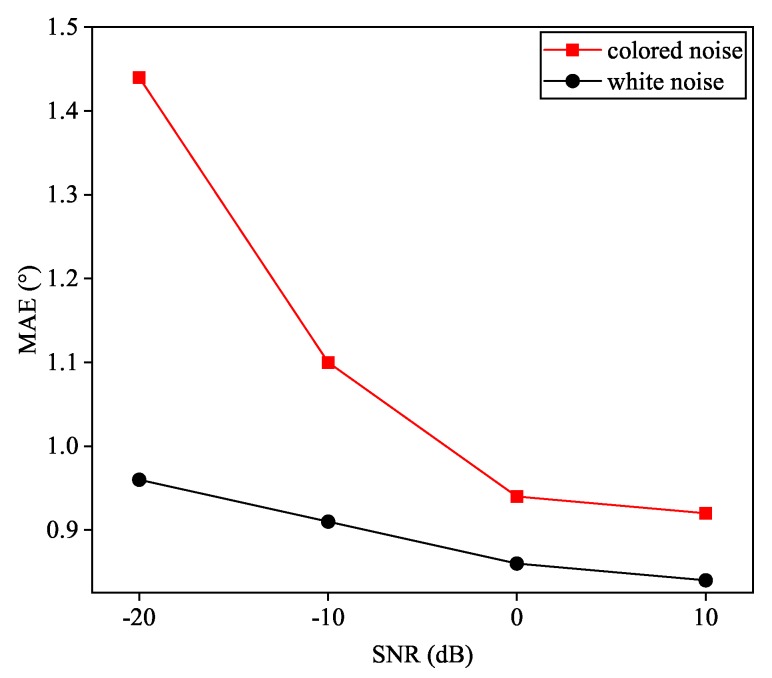
Performance of the Toeplitz A-CRNN models to colored noise while trained in AWGN.

Figure 12 gives out the testing results of proposed Toeplitz A-CRNN network to the signals from three coherent sources contaminated with colored Gaussian noise. Our models are trained in white Gaussian noise at the SNR of 20 dB, and are tested at −20 dB, −10 dB, 0 dB, and 10 dB. Estimation accuracies in the untrained colored-Gaussian-noise scenario approximately go through a 0.5° decline in terms of the MAE measurement.

### 4.3. Discussions

As shown in above numerical experiments, the Topelitz A-CRNN method outperforms baselines in fitting the mapping from array received data to arrival directions, because BiLSTMs in CRNN units can adequately explore the time-dependency among local features extracted by CNNs. Owing to the Topelitz module in the proposed framework, the trained network can be self-adaptive to changeable number of sources, which even are coherent.

Besides the performances, we also need to analyze the testing computational complexities. As for the FC-NN algorithm [19], the complexity is $O(L_{in}L_{out})$, where *L* denotes the size of layer input or output vector. Computations in SS-MUSIC are mainly generated from subspace decomposition and spectral peak-searching and can be formulated as $O({\left(2M+1\right)}^{2}S+{\left(2M+1\right)}^{2}F)$ [31], where *F* is the number of spatial frequencies which is related to the resolution. In the proposed network, the heaviest calculation burden arises from the BiLSTM part, which is O(W). *W* is the number of parameters in BiLSTM layers, which is always far greater than $L_{in}L_{out}$.

## 5. Conclusions

In summary, this work demonstrates a new structure of DL network to address the DOA estimation problem. Designed spatial filters and alternate multi-label classifiers based on CRNN units can recover the arrival angles of coherent signals. With the facilitation of Toeplitz matrix reconstruction, our framework still reaches a high estimation accuracy when the source number is unknown. Simulations on ULA show great advantages to state-of-the-art FC-NN and the conventional SS-MUSIC algorithm especially when arrival directions of the sources are adjacent. Meanwhile, our trained Toeplitz A-CRNN model reveals excellent adaptation to practical conditions such as limited snapshots, array imperfections, lower SNR, and colored Gaussian noise. In addition, as networks can be trained offline, the proposed method is computational efficient in real-time testing phase, which shows good prospects for realistic applications.

Further studies to extend the proposed A-CRNN framework can be making it adaptable to different kinds of input-signals and array geometries. We will consider to introduce the multimodal learning strategy (see, e.g., [32,33]) to the framework for potential realization.

## Figures and Tables

**Table 1:** Parameters in regard to simulation conditions.

Parameter	Description	Value
2M+1	number of array sensors	11
$\left(\theta_{min},\theta_{max}\right]$	angular range	(−60°,60°]
Δθ	angular resolution	1°
*L*	number of sectors	6
*S*	number of snapshots	100

**Table 2:** Settings for training the spatial filters and multi-label classifiers on Keras.

Item	Spatial Filter	Multi-Label Classifier
network sturcture	32-CRNN unit	128-CRNN unit
		+64-CRNN unit
loss function	Focal	*binary-crossentropy*
epochs	50	100
noise-signal ratio	20 dB	
size of mini-batch	50	
regularization	$l_{1}$-norm	
optimizer	Adam	

**Table 3:** Accuracies of three-source coherent DOA estimation at the SNR of 20 dB.

	Absolute Error				Operation Time
Models	<1°	<4°	<7°	<10°	(s)
FC-NN	27.29%	46.03%	49.44%	51.16%	**0.00049**
SS-MUSIC	82.17%	82.41%	83.05%	87.36%	0.0049
A-CRNN	87.39%	89.14%	90.35%	91.38%	0.0012
Toeplitz A-CRNN (GRU)	93.90%	96.06%	96.85%	97.30%	0.0010
Toeplitz A-CRNN (proposed)	**95.77**%	**97.58**%	**98.15**%	**98.46**%	0.0012
